# The Relationship between Thyrotropin Serum Concentrations and Thyroid Carcinoma

**DOI:** 10.3390/cancers15205017

**Published:** 2023-10-17

**Authors:** Xueqi Zhang, Lijun Tian, Di Teng, Weiping Teng

**Affiliations:** Department of Endocrinology and Metabolism, Institute of Endocrine, NHC Key Laboratory of Diagnosis and Treatment of Thyroid Diseases, The First Hospital of China Medical University, Shenyang 110001, China; zxq1823@163.com (X.Z.); tianlijun0910@163.com (L.T.); twp@vip.163.com (W.T.)

**Keywords:** tyrotropin, serum concentrations, TSH, thyroid cancer, epidemiological studies, prognosis

## Abstract

**Simple Summary:**

Thyroid-stimulating hormone (TSH) plays a role in the regulation of thyroid hormones and is an important indicator for assessing thyroid function. Thyroid cancer (TC) is a common tumor of endocrine system, and its incidence rate is increasing year by year. The role of TSH in the thyroid gland suggests a potential influence on the occurrence and progression of TC, which has attracted the attention of the scientific community. Based on the controversial fact that TSH may affect TC, this article reviews the epidemiological evidence and potential mechanisms of the relationship between TSH and TC, in order to provide valuable references for future research work.

**Abstract:**

Thyroid Stimulating Hormone (TSH) is a hormone secreted by the pituitary gland and plays a role in regulating the production and secretion of thyroid hormones by the thyroid gland. This precise feedback loop is essential for maintaining a harmonious balance of thyroid hormones in the body, which are vital for numerous physiological processes. Consequently, TSH serves as a significant marker in assessing thyroid function, and deviations from normal TSH levels may indicate the presence of a thyroid disorder. Thyroid cancer (TC) is the malignant tumor within the endocrine system. In recent years, numerous experts have dedicated their efforts to discovering efficacious biomarkers for TC. These biomarkers aim to improve the accurate identification of tumors with a poor prognosis, as well as facilitate active monitoring of tumors with a more favorable prognosis. The role of TSH in the thyroid gland underscores its potential influence on the occurrence and progression of TC, which has garnered attention in the scientific community. However, due to the limited scope of clinical research and the dearth of high-quality foundational studies, the precise impact of TSH on TC remains unclear. Consequently, we present a comprehensive review of this subject, aiming to offer a valuable reference for future research endeavors.

## 1. Introduction

Thyroid cancer(TC) is a type of cancer in the endocrine system [1]. The primary treatment involves surgical intervention. Post-operatively, radioactive iodine therapy may be administered depending on the circumstances, followed by hormone replacement therapy [2,3]. The most common type of TC is papillary thyroid cancer (PTC), accounting for about 80% of all TC and usually grows slowly and has a good prognosis [4]. Follicular thyroid carcinoma (FTC) is less common, accounting for about 10–15% of all TC, tends to invade blood vessels, and has a higher risk of metastasis compared to PTC. Medullary thyroid carcinoma (MTC) is a rare type of TC that originates from the C cells of the thyroid gland, which accounts for about 5–10% of all TC and has a higher growth rate and metastasis rate compared to PTC and FTC. Anaplastic thyroid carcinoma (ATC) is the least common type, but it is the most aggressive and dangerous [5]. Indeed, with the progress of diagnostic technology and the growing focus on health, it is widely recognized that the high incidence of TC is largely attributed to adequate diagnosis. Improved techniques, such as ultrasound and fine-needle aspiration biopsy, have enhanced the detection of small and indolent thyroid nodules that may have previously gone unnoticed [6]. This phenomenon has sparked discussions regarding the appropriate management of these indolent cases, underscoring the importance of identifying crucial markers to accurately identify high-risk TC [7,8,9,10,11,12,13,14]. In recent years, medical experts have been dedicated to finding effective prognostic markers for PTC, such as radiation exposure, iodine nutrition [15], thyroid hormone, thyroid stimulating hormone(TSH), BMI [16], metabolic syndrome [17], gene mutations [18], plasma exocrine markers, among others. Unremitting research and exploration of these biomarkers can also help identify low-risk papillary thyroid microcarcinoma (PTMC), enabling active surveillance (AS) and avoiding overtreatment [19,20].

As an important member of the hypothalamus–pituitary–thyroid axis in the endocrine system, TSH plays a crucial role in regulating the growth, differentiation, and function of thyroid cells [21]. Therefore, it has always been considered closely related to TC. Currently, most studies have compared the TSH levels of TC patients and individuals with benign thyroid disease or healthy controls. These findings from various studies indicated that TC patients often demonstrate elevated TSH levels, even when their TSH falls within the normal range. However, it is important to highlight that the current evidence primarily relies on cross-sectional studies, which mainly describe a phenomenon without establishing causal relationships. The involvement of TSH in the early onset or late progression of TC remains unknown, the interaction between TSH and other factors and how it affects TC is not well understood [22].

We summarize the current research status in this field, aiming to elucidate the relationship between TSH and TC, and provide guidance and direction for future research endeavors in this area.

## 2. Methods of Literature Research

We conducted a systematic literature search in PubMed, Embase, Cochrane Library, and Web of Science databases until July 2023. The search was conducted by using the search terms such as “thyroid stimulating hormone”, “TSH”, and “thyroid cancer”. We selected relevant articles by classifying the title, abstract, and full text of all the studies and limiting the included studies to those published in English. Two researchers independently selected articles and reviewed the abstracts and full text of the articles.

## 3. Epidemiological Studies

### 3.1. Effect of TSH on the Prevalence of TC

Previous studies have provided evidence that the TSH levels in TC patients are significantly higher. For example, Jonklaas et al. conducted a clinical study in 2008 and found that TSH levels in TC patients were significantly higher than those in benign diseases, and even within the normal range, high TSH concentrations were associated with the diagnosis of TC in patients. They also indicated for the first time that patients with TC have lower triiodothyronine (T_3_) levels than patients with benign disease [23]. In 2009, Fiore et al. analyzed the relationship between TSH and the occurrence of PTC in 10,178 patients with fine needle aspiration cytology or benign thyroid nodule diseases. They also indicated that the incidence of PTC increased with the increase in TSH, while the occurrence of autonomous thyroid function (TSH < 0.4 mU/L) is associated with a reduced risk of PTC [24,25]. Kim et al. investigated whether serum thyroid antibodies can predict the occurrence of TC in patients with thyroid nodules, and found that in 1638 patients, thyroglobulin antibody (TgAb) and TSH levels (2.5 ± 2.8 mU/L vs. 2.1 ± 2.0 mU/L) in TC were higher than those in benign nodules. Their results verified that the upper third of the normal range of TSH levels and TSH levels above the normal range were risk factors for TC [26]. In 2011, Zafon et al. evaluated the level of TSH in 386 patients with nodular thyroid disease before an operation and discovered that the average concentration of TSH in patients with benign diseases was 1.36 ± 1.62 mU/L and that in patients with malignant diseases was 2.08 ± 2.1 mU/L. In malignant patients, the average TSH of PTMC and thyroid cancer of larger size (TCLS) was 1.71 ± 1.52 mU/L and 2.42 ± 2.5 mU/L, respectively [27]. In summary, the relationship between TSH, PTC, and tumor size has been revealed. In 2012, our team conducted a retrospective study on postoperative pathology of 1870 patients who underwent surgery on thyroid nodules. We found that elevated TSH levels were associated with lymph node metastasis (LNM) and advanced disease in cases of differentiated thyroid cancer (DTC). However, this pattern of higher TSH levels correlating with a higher incidence of disease did not apply to microcarcinomas of the differentiated thyroid microcarcinoma (DTMC) [28]. In 2013, Ye et al. investigated the risk factors of PTC and found that the risk of PTC increased simultaneously with an increase in TSH concentration within the normal range. Compared with TSH below 0.35 mIU/L, the risk of malignant tumors was significantly increased when serum TSH was between 1.97 and 4.94 mIU/L [29]. Another study in children revealed that malignancy in patients with nodules is most likely to occur at the upper normal limit of TSH (>2.8 mIU/L), and the serum TSH concentration in patients with TC is significantly higher than that in patients with benign nodules (3.23 ± 1.95 mU/L vs. 1.64 ± 0.99 mU/L), once again confirming the value of TSH [30]. In 2014, to determine whether TSH levels play a predictive value for malignancy in patients with nodules, Zeng et al. surveyed 108 individuals and found that the average TSH value in malignant patients was higher than that in benign patients, with TSH levels of 1.94 ± 1.01 mU/L and 1.16 ± 0.85 mU/L, respectively. Compared with patients with TSH levels below the population average, patients with TSH levels higher have a significantly higher incidence of malignant tumors (35.9% vs. 15.9%) [31]. A meta-analysis in 2022 suggested that an increase in TC risk is associated with high TSH exposure (OR = 1.28), and for every 1 mU/L increase in TSH, TC risk increases by 16% [32]. Other epidemiological studies and meta-analyses have also investigated the pathological data and TSH levels of PTC patients, revealing the relationship between TSH exposure and PTC, and providing evidence that TSH may promote PTC [33,34,35,36,37]. Results from other research also suggested that the impact of TSH on TC may vary depending on the histological type. For example, one study in 27,914 patients showed that in patients with thyroid nodules, reducing TSH levels through levothyroxine replacement therapy mainly helps to reduce the recurrence of PTC [38]. A meta-analysis in 2016 summarized 22 related articles and found that serum TSH was associated with an increased risk of TC, with the most significant impact on PTC. Conversely, high TSH levels were associated with a reduced risk of FTC. Therefore, future research needs to focus on the histological types of TC [39].

Most of the above were retrospective studies with limited evidence, while a prospective study in 2017 confirmed the above conclusions. A total of 615 patients participated in this study, and ultimately, patients with malignant tumors had higher TSH levels than patients with benign nodules. Patients with TSH levels ≥ 2.26 mU/L have a risk of developing malignant tumors approximately three times higher than those with low TSH levels [40]. TSH levels may play an auxiliary diagnostic function to stratify malignant tumors and may help determine the optimal treatment method.

While TSH has significance in the diagnosis of some thyroid diseases, relying solely on TSH levels is not sufficient to exclude TC in all patients with functional thyroid nodules [41]. For example, a study in 2017 concluded that there is a significant gender difference in the impact of TSH on PTC, meaning that TSH levels above the normal range are only associated with an increased risk of developing PTC in males, while TSH levels are relatively low in female PTC patients [42]. In addition, another study showed a positive correlation between TC risk and Tg levels and a negative correlation with TSH levels, indicating that low TSH levels may lead to the occurrence of TC [43]. Although Sohn et al. verified that the frequency of PTC increased with the increase in serum TSH, it is only applicable to large nodules > 1 cm and is not helpful for clinical risk assessment of small thyroid nodules [44]. The latest evidence came from a large cohort study conducted by Kim et al. in South Korea in 2022 [45]. They found that when comparing low-level TSH with normal-level TSH in men or women, the adjusted hazard ratio (HR) was 2.95, 1.5 in men and women, respectively, which means that within the normal range of thyroid function, the TC risk of the highest three quantiles of TSH is lower than that of the lowest three quantiles of TSH.

However, some studies have shown that there is no correlation between TSH levels and the risk of developing PTC. A meta-analysis in 2012 evaluated the incidence of TC in patients with nodular goiter undergoing surgical treatment and did not confirm the association between TSH and TC, which indicates that larger prospective studies are still warranted to verify this point [46]. Wang et al. conducted a retrospective study in 2021 that evaluated the correlation between TSH concentration and characteristics of pediatric TC [47]. In the univariate analysis, serum TSH levels were not correlated with thyroid nodule malignancy but were negatively correlated with thyroid nodule malignancy after adjusting for gender, age, thyroid autoantibody status, and nodule size (OR = 0.856). So, serum TSH concentration does not seem to be a carcinogenic factor for pediatric thyroid nodule patients, nor was it an independent risk factor for characteristics of existing pediatric TC.

The main findings of the research were shown in Table 1. Due to the differences in current conclusions, further research is needed to reveal its true role in the occurrence of TC and its potential mechanisms (Table 1).

### 3.2. The Effect of TSH on the Invasiveness of TC

Several studies have shown a correlation between TSH levels and the progression of TC, making it possible to control the progression of TC through the suppression of TSH for patients, but it remains a topic of ongoing debate.

Currently, various studies have confirmed the association between high TSH levels and TC invasiveness [48,49]. For example, a retrospective cross-sectional analysis in 2010 compared the average TSH levels of PTMC patients and found a positive correlation between tumor size and TSH levels. They also proposed that TSH is unlikely to be involved in the occurrence of PTMC, but may play a role in the progression of existing PTMC [50]. To investigate whether TSH concentration can be used for risk prediction of DTC, especially DTMC, our team conducted a retrospective study investigating the serum TSH levels of 1870 patients undergoing thyroid nodule surgery in hospitals in iodine-rich areas of China [28]. It was found that the incidence of DTC significantly increased with the increase in TSH; higher TSH is also associated with LNM and advanced diseases (stages III and IV). However, the pattern of increased incidence of elevated TSH does not apply to DTMC. Overall, serum TSH is not a good risk predictor for DTMC, but elevated TSH levels may be associated with the progression of TC. In 2014, McLeod et al. tried to assess whether preoperative serum TSH and TgAb in patients with TC were related to prognosis. They analyzed the tumor stage, persistent disease, recurrence, and overall survival of 617 patients with preoperative serum TSH and 1770 patients with perioperative TgAb status, and found that preoperative serum TSH level was related to higher tumor stage, extrathyroidal extension, and cervical LNM [51]. A multicenter retrospective study in 2016 measured the TSH concentration of patients, and an increase in TSH levels was found to be associated with thyroid extension and LNM [49]. In the same year, Gao et al. demonstrated that serum TSH levels ≥ 2.5 mIU/L were an independent predictor of LNM in 162 PTMC patients, once again confirming this conclusion [52]. Dumont et al. demonstrated that compared to benign diseases, elevated TSH levels increase the risk of PTC and affect its prognosis, especially in promoting LNM [53]. Subsequently, Tam et al. conducted a retrospective study in 2018 and analyzed the relationship between preoperative serum TSH levels and clinical pathological characteristics in patients with PTC and PTMC [54]. The results showed that benign nodular diseases had the lowest serum TSH, higher PTMC, and highest PTC. Compared with patients with unilateral tumors, patients with bilateral tumors, capsule invasion, and LNM have higher serum TSH, and invasive PTC patients have higher serum TSH than noninvasive PTC patients. In 2022, Kim et al. found during AS of PTMC that TSH is the key factor in PTMC progression, but its impact is only significant in patients under 50 years old, which reminded us that lower TSH levels during AS may help prevent progression [43].

Other studies have found that TSH may not have a role in the progression of TC. In the 5.6-year follow-up conducted by Kim et al., 21 patients (1.4%) experienced recurrence, and 38.1% or 36.0% of patients had average TSH levels within the recommended low normal range (0.5 to 1.9 mIU/L) throughout the entire follow-up period or 5 years, indicating that serum TSH levels did not affect short-term recurrence in low-risk DTC patients after thyroidectomy [55]. Similarly, a study conducted in 2020 with 3973 participants found that in the presence of thyroid autoimmunity, there was no significant association between TSH concentration and the risk of developing DTC [56].

### 3.3. The Combined Effects of TSH and Other Factors on TC

The relationship between TSH and TC is complex, and there may be interactions with other risk factors that contribute to the development of TC. For example, exposure to ionizing radiation [57], genetic mutations [58], the levels of serum thyroid peroxidase antibody (TPOAb) or TgAb [59], environmental factors, and underlying thyroid diseases may also increase the risk of developing TC. As an important endocrine hormone, exploring the association of TSH and other factors in clinical research will help to understand the pathogenesis of TC.

The results of Kim et al. in 2010 suggested that the positive rate of TPOAb in malignant tumors is not high, while TgAb positivity and high TSH levels are significantly associated with TC [26]. In a retrospective study conducted by Wu et al. in 2014, it was verified that compared to TgAb or TPOAb positivity alone, the simultaneous positivity of TgAb and TPOAb confers a greater risk of PTC and is associated with increased TSH levels and advanced PTC stages, further suggesting that the high TSH phenomenon in PTC may be related to thyroid autoimmunity [60]. In 2016, the results of a prospective cohort study showed that preoperative assessment of serum Tg and TSH levels did not seem to help identify TC patients. However, a higher ratio of preoperative serum TSH to Tg may indicate an increased risk of TC [61]. In 2019, Hu et al. investigated the correlation between fasting serum glucose (FSG), TSH, FT_3_, FT_4_, and PTC and found a significant correlation between higher TSH levels and the occurrence of PTC, especially PTMC. This study also demonstrated that the occurrence of PTC is negatively correlated with serum FT_3_ levels and positively correlated with FT_4_ and FSG levels [62]. In addition, Jonklaas et al. explored the relationship between serum selenium concentration and TC diagnosis in selenium-rich areas of the United States. They found a relationship between serum TSH and selenium concentration with advanced TC, and future research needs to determine whether there is a potential connection between selenium and TSH to affect the progression of TC [63].

Overall, the interaction between TSH and multiple factors may play a role in the development and progression of TC. Further research is needed to better understand these complex interactions and determine strategies for accurately identifying high-risk TC patients.

### 3.4. The Effect of TSH Inhibition on TC

In past decades, the diagnosis and management methods of TC have made continuous progress. The main treatment for reducing TSH levels in the blood is to use levothyroxine (LT_4_) as an alternative, which is suitable for the preoperative and postoperative treatment of TC patients [64,65]. It is widely believed that TSH inhibition therapy may reduce the proliferation and survival ability of TC cells, thereby reducing bleeding and pain. Postoperative TSH suppression therapy is believed to reduce the risk of TC recurrence and metastasis. TSH suppression therapy in TC patients is based on several factors, including the stage and type of cancer, the patient’s age, and overall health. The effectiveness of this treatment has not been widely recognized, and its safety is still controversial [66,67].

For example, Park et al. conducted a cohort study with a median follow-up period of 8.6 years and found that among low-risk DTC patients undergoing thyroidectomy, TSH suppression therapy had no clinical benefits, which indicated that LT_4_ is not necessary for patients without evidence of hypothyroidism [68]. Similarly, in 2019, Gwiezdzinska et al. followed up on a group of medium to high-risk DTC patients to evaluate the association between TSH inhibition and progression-free survival and overall survival, but the results implied that medium to high-risk DTC patients may not benefit from TSH inhibition treatment [69].

There are many negative outcomes related to active TSH suppression therapy, including osteoporosis, fractures, and cardiovascular diseases [66,70,71,72]. For example, a 2019 meta-analysis applied a significant decrease in bone density in DTC patients who received TSH inhibition treatment, suggesting that attention should be paid to the long-term bone safety of DTC patients [73]. Lee et al. evaluated the parameters related to muscle mass and muscle function in DTC patients who received TSH inhibition treatment after surgery and found that muscle function should be considered an adverse reaction to TSH inhibition in DTC patients, especially males < 70 years old [74]. In addition, there are still some issues with the application of L-T_4_, such as the varying degrees of application of TSH inhibition therapy by clinical doctors, and the recommendations of L-T_4_ for treating DTC patients have not been properly implemented in clinical practice, resulting in immature TSH inhibition strategies [74,75].

According to the 2015 guidelines from the American Thyroid Association (ATA), when TSH levels are higher than 2 mU/L, there is an increased risk of TC-related mortality and recurrence. For high-risk DTC patients, it is recommended that TSH be suppressed to below 0.1 mU/L postoperatively, which significantly reduces the risk of tumor recurrence and metastasis. In the case of non-high-risk DTC patients, postoperative TSH suppression to the range of 0.1 to 0.5 mU/L can significantly improve the overall prognosis. However, for non-high-risk DTC patients, further suppressing TSH to below 0.1 mU/L does not provide additional benefits. The benefits of TSH suppression therapy may be limited for low-risk DTC patients with a low risk of recurrence. In addition to the above guidelines, we also need to take into account that TSH levels tend to naturally increase with age, but currently, there are no age-specific reference ranges for TSH [76,77]. Considering the physiological changes in older individuals, it is necessary to appropriately relax the control of TSH levels in the elderly.

This further emphasizes the importance of TSH suppression in DTC treatment and underscores the significance of individualized treatment strategies, particularly in tailoring treatment plans based on the patient’s risk profile.

## 4. Research on Mechanisms

While many studies have shown a correlation between TSH and the development of thyroid cells and TC, the exact mechanisms by which TSH promotes this process are still not completely understood [78]. Mitsumori et al. found that the TSH level in the early stage of FTC in rats increased rapidly, which supported the conclusion that high serum TSH levels played an important role in the early stage of FTC [79]. In 2001, a study collected tissues from 13 patients undergoing TC surgery for immediate culture and incubated cells with different concentrations of TSH. They found that the proliferation rate of TC cells was not affected by TSH levels [80]. To identify the sequence variants with a risk of TC, Gudmundsson conducted a genome-wide association study in 192 and 37,196 Icelandic cases and controls, respectively. They observed the strongest correlation signal of rs965513 on 9q22.33 (OR = 1.75; *p* = 1.7 × 10^−27^) and rs944289 on 14q13.3 (OR = 1.37; *p* = 2.0 × 10^−9^), both of which contribute to an increased risk of TC, estimate that the risk of TC is 5.7 times that of noncarriers and in their large sample set targeting the general population, both risk alleles were associated with low concentrations of TSH [81]. In 2009, Friedman demonstrated that CD133^+^ATC cells could initiate tumors in immunodeficient mice, while the process was regulated by TSH [82]. In 2010, Lu et al. conducted an experiment using a mouse model of FTC and found that the stimulation of TSH was a prerequisite for the development of metastatic cancer, but this is not enough to lead to TC [83]. In 2014, a study found that TSH stabilized the dual specific phosphatase 6 (DUSP6) protein by increasing the expression of Mn superoxide dismutase (MnSOD), and inhibited BRAF-induced senescence. TSH, together with DUSP6, reactivated the RAS signaling pathway, leading to the activation of RAS/AKT/glycogen synthase kinase 3β and stabilizing the C-Myc protein by inhibiting its degradation [84]. Currently, research on the mechanism of the association between TSH and TC mainly involves thyroid hormone receptors (TSHR), major gene mutations, and cell proliferation-related signaling pathways. We summarize them in the following text.

TSHR, a kind of thyroid-specific protein, was considered a differentiation marker of thyroid follicular cells [85]. When TSH binds to TSHR, it stimulates the thyroid gland to produce and release thyroid hormones into the bloodstream, which are vital for regulating metabolism, growth, and development throughout the body. In 1995, Ohno et al. discovered structural changes in TSHR in human TC tissue [86]. Considering the crucial role of TSHR, Xing et al. investigated the potential molecular mechanisms that affect TSHR expression and found frequent CpG methylation in TC, while no methylation was found in normal thyroid tissue and benign adenomas [87]. In human TC cells, they observed that TSHR is usually expressed at the level of protein and mRNA in cells with unmethylated TSHR gene, while it was silenced in cells with hypermethylated TSHR promoter. After treatment with demethylating agents, a cell partially restores TSHR expression. Therefore, it is suggested that methylation of TSHR may provide new diagnostic biomarkers for malignant tumors. Another study explored the relationship between TSHR and TC recurrence, which revealed that compared with the primary site, the expression of TSHR in the recurrent site is lower, which is closely related to the poor prognosis of TC patients, significantly reducing disease-free interval and shortening overall survival [88]. In 2022, Wu et al. once again demonstrated that the TSH-TSHR Axis promotes tumor escape in thyroid cancer and glioma, indicating that TSH suppression therapy is an effective treatment strategy for immune checkpoint blockade combined therapy [89]. At present, it is unclear whether long-term TSH stimulation can promote KRAS ^G12D^- mediated transformation of thyroid follicular cells. Therefore, Zou et al. studied the effect of long-term TSH stimulation on KRAS^G12D^ knock-in mice and the role of Sprouty1 (SPRY1) in KRAS^G12D^-mediated signal transduction and found that chronic TSH stimulation can enhance KRAS^G1D^-mediated tumorigenesis, leading to FTC [90].

Additionally, BRAF plays an important role in the pathogenesis and prognosis prediction of TC, activating the mitogen-activated protein kinase (MAPK) pathway and leading to increased cell proliferation, dedifferentiation, and apoptosis, which has been confirmed before [91]. In 2011, Franco et al. explored the mechanism of the occurrence of TC by establishing a mouse model with a thyroid-specific knock-in of oncogenic BRAF and found that the activity of the TSH signaling pathway in mice can promote BRAF mutations in thyroid cells, which involves the MAPK signaling pathway [92]. Another supporting evidence was that Zou et al. experimented in mice that oncogene-induced senescence (OIS) is activated in BRAF-induced PTC tumors when serum TSH is normal, and this process depends on normal p53 function [93]. Meanwhile, TSH can reduce the expression of p53 and inhibit OIS by upregulating the PI3K/AKT pathway, leading to tumor progression. These data indicated that TSH has an important carcinogenic effect in the progression of thyroid tumors. Through long-term TSH stimulation, TC cells can gradually become TSH-independent cells and progress to poorly differentiated cancer. TSH may promote the proliferation of thyroid cells by activating various signaling pathways.

In 2016, our team found that TSH-induced proliferation of TC cells relies on the transduction of TSHR/cAMP/PKA/PAK4 signaling, revealing a new function of TSH and PAK4 in TC progression [94]. In addition, TSH and insulin/IGF-I mainly synergistically induce thyroid cell proliferation through the cAMP and phosphatidylinositol 3-kinase (PI3K) pathways. Zaballos found that Forkhead box O (FoxO)-1 can play an important role in the influence of TSH and IGF-I on thyroid cell proliferation, indicating that the loss of FoxO1 expression is related to the uncontrolled proliferation of TC cells [95].

Vascular endothelial growth factor (VEGF) has been proven to be the most important endothelial mitogen, but the effect of TSH on VEGF production has not been studied. Hoffmann et al. conducted in vitro experiments using HTC, a follicular cancer cell line lacking endogenous TSHR and its receptor-positive variant (HTC TSHR), and Huerthle cell-derived (XTC) cell lines in 2004 [48]. They evaluated the levels of VEGF-related proteins under basic and TSH stimulation conditions to determine TSHR signal transduction and found that TSH increased VEGF mRNA and protein in HTC TSHR and XTC cells by 40% in a dose-dependent manner. It was verified that in TC cell lines, TSH can induce VEGF production involving the PKC rather than the PKA pathway. In 2019, Song et al. further discussed the impact of TSH on the microenvironment of PDTC tumors [96]. After treatment with TSH, they found that abnormal TSHR signal transduction increased the secretion of VEGF-A and CXCL8 in TC cells, thereby promoting angiogenesis and tumor growth.

**Figure 1:**
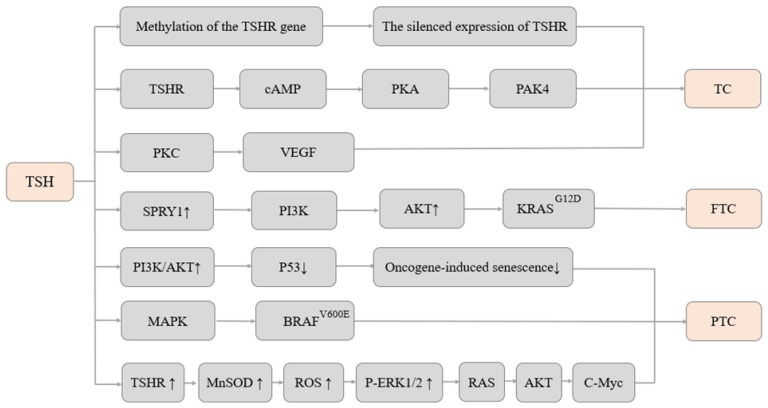
Research status on the mechanism of the association between TSH and TC. ↑: up-regulated; ↓: down-regulated.

Overall, the mechanisms by which TSH promotes the development of thyroid cells and TC are likely to be complex and multifactorial, and further research is needed to fully understand these processes. The summary of these mechanisms can be seen in Figure 1.

## 5. Conclusions

Various epidemiological studies have revealed a positive association between high levels of TSH and an increased risk of TC. Some studies have also provided evidence of malignancy in TSH and TC patients, such as invasiveness, LNM, etc. This may be because TSH stimulates the growth and division of thyroid cells, and long-term exposure to high levels of TSH may increase the risk of genetic mutations and other changes in the thyroid cells, leading to the development of TC. Meanwhile, some studies have drawn opposite conclusions. Considering these viewpoints, we believe that current research has certain limitations, such as the correlation between TSH and TC being mainly cross-sectional and unable to explain their causal relationship. Therefore, it is not possible to determine whether TSH has a stimulating or inhibitory effect on TC based on the existing evidence. In addition, most of the research data come from the test results of preoperative TC patients, and there is a lack of large-scale prospective studies conducted in the general population, which may have poor representativeness. Indeed, due to the lower degree of differentiation of TC, the secretion of T3 may decrease. This reduction in T3 secretion can lead to an increase in TSH levels through a feedback mechanism. This may elucidate the rationale behind elevated TSH levels in TC. It suggests that higher TSH levels in TC may not be the cause but rather a consequence.

The differences in pathological characteristics and biological behavior between TC do indeed influence their response to TSH. For example, PTC typically exhibits a lower degree of differentiation, meaning its cellular structure is relatively dissimilar to that of normal thyroid cells. This may lead to a relatively higher sensitivity to changes in TSH levels due to the lower degree of differentiation, making PTC cells more responsive to TSH fluctuations. Additionally, PTC is often accompanied by elevated levels of Thyroglobulin (Tg). This may impact the regulation mechanism of TSH, as the increase in Tg levels may in some way influence the thyroid’s feedback mechanism. However, the specific reasons and mechanisms may require further research for clarification. Furthermore, factors such as individual variations, sample size, and research methodologies can also impact the interpretation of clinical and experimental data. Therefore, caution should be exercised when interpreting study results.

TSH suppression therapy has emerged as a prevalent option for post-surgical TC patients. However, existing evidence does not unequivocally support the benefits of this therapy, and it may carry potential side effects, including palpitations, cardiovascular disease, anxiety, and bone loss. Consequently, it is imperative to delve deeper into the efficacy of TSH inhibition therapy, adopt rational and standardized strategies for its implementation, and individualize patient management based on their specific conditions. Close monitoring of TC patients undergoing TSH inhibition therapy by their healthcare providers is of utmost importance.

Currently, research on the mechanisms of TSH and TC is continually deepening. Some studies are investigating the role of TSHR, key molecules, and regulatory factors in the TSHR signaling pathway in TC. The primary focus of these studies has been to explore the association between TSH and TC metastasis, as well as to discover novel treatment approaches. Additionally, TSH may also impact the growth and metastasis of TC through various signaling pathways such as PI3K/AKT and MAPK pathways. However, the available research evidence is still limited, and the relationship between TSH and TC is complex and diverse. Further research is needed to unveil the underlying mechanisms and provide better insights and methodologies for the treatment of TC.

## Figures and Tables

**Table 1:** The main characteristics of the studies on the impact of TSH on the prevalence of TC.

No.	First Author	Publication Year	Location	Main Findings
1	Jonklaas et al. [23]	2008	American	Compared to patients with TSH concentrations in the lowest 1/4, patients with TSH concentrations in the upper 3/4 have a higher risk of TC (OR = 8.7).
2	Fiore et al. [25]	2009	Italy	The TSH levels of PTC are significantly higher than those of benign thyroid diseases and are highest among patients with serum TSH at the upper limit of the normal range. (TSH > 3.4 mU/L, OR = 3.5).
3	Kim et al. [26]	2010	Korea	Benign nodules: 2.1 ± 2.0 mU/L; TC: 2.5 ± 2.8 mU/L Upper 1/4 of TSH levels: OR = 1.72; above normal range of TSH levels: OR = 1.98
4	Zafon et al. [27]	2011	Spain	Benign nodules: 1.36 ± 1.62 mU/L; TC: 2.08 ± 2.1 mU/L PTMC: 1.71 ± 1.52 mU/L; TCLS: 2.42 ± 2.5 mU/L
5	Ye et al. [29]	2013	China	The risk for malignancy significantly increased with serum TSH 1.97–4.94 mIU/L, compared with TSH less than 0.35 mIU/L (OR = 1.951)
6	Mussa et al. [30]	2013	Italy	Benign nodules: 1.64 ± 0.99 mU/L; TC: 3.23 ± 1.95 mU/L
7	Zeng et al. [31]	2014	China	Benign tumors: 1.94 ± 1.01 mU/L; PTC: 1.16 ± 0.85 mU/L
8	Rinaldi et al. [43]	2014	France	TC risk was negatively associated with TSH level (OR = 0.56)
9	Huang et al. [42]	2017	American	TSH levels below the normal range were associated with an elevated risk of PTC among women (OR = 3.74); TSH levels above the normal range were associated with an increased risk of PTC among men (OR = 1.96).
10	Golbert et al. [40]	2017	Brazil	Patients with TSH levels ≥ 2.26 mU/L have a risk of developing malignant tumors approximately three times higher than those with low TSH levels.

## Data Availability

The data can be shared up on request.
